# Fatigue and Exercise Intolerance as Initial Manifestations of a Nonsyndromic Mitochondrial Disorder Due to the Variant m.3243A>G

**DOI:** 10.1155/2022/7846852

**Published:** 2022-03-23

**Authors:** Josef Finsterer, Sinda Zarrouk

**Affiliations:** ^1^Neurology and Neurophysiology Center, Vienna, Austria; ^2^University of Tunis El Manar, Tunis, Tunisia

## Abstract

**Objectives:**

Fatigue and exercise intolerance have been only rarely reported as initial- and sole-onset manifestations of a mitochondrial disorder (MID). We present a patient with nonsyndromic MID with fatigue and exercise intolerance as its initial manifestations of the disease. *Case Report*. A 39 yo female experienced fatigue since age 18 and exercise intolerance since age 21. Later on, she developed Hashimoto thyroiditis, recurrent diffuse headache, and double vision upon exercise. Clinical exam revealed short stature, bilateral ptosis, partially reduced tendon reflexes, and hypertrophic calves. Serum lactate was elevated, and the lactate stress test was abnormal. Workup for suspected MID revealed ragged-red fibers and NADH-deficient muscle fibers, and biochemical investigations revealed a mild complex-I defect. mtDNA sequencing revealed the variant m.3243A>G with a heteroplasmy rate of 70% in the muscle.

**Conclusions:**

This case shows that the initial manifestation of a MID can be fatigue and exercise intolerance. MIDs due to the m.3243A>G variant may have a slowly progressive course and only delayed multisystem involvement. The variant m.3243A>G may not only manifest as syndromic MID, particularly MELAS but also as nonsyndromic phenotype. MIDs should be considered as differentials of chronic fatigue even if no other phenotypic manifestation of a MID is present.

## 1. Introduction

Mitochondrial disorders (MIDs) can be due to mutations on the mtDNA or in nDNA located genes [1]. They manifest with a broad range of phenotypic features and have an onset between birth and senescence. Though fatigue is a common phenotypic feature of MIDs [1], it has been only rarely reported as the initial and sole manifestation of a MID. Usually, fatigue is one feature among others at onset or develops later in the course. Here, we present the data of a MID patient with fatigue and exercise intolerance as the initial manifestations of the disease. Informed consent was obtained from the patient.

## 2. Case Report

The patient is a 39 yo female, height 160 cm, with an uneventful previous history until age 18 y when she started to recognise increased tiredness and fatigability. At age 21 y, she developed exercise intolerance initially only during sport activities but later also in association with simple activities of daily living. Overexertion was complicated by nausea and tiredness. Later in the course, Hashimoto thyroiditis was diagnosed and hypothyroidism substituted with L-thyroxin, without a beneficial effect on fatigue. During the further course, she developed episodic nonmigrainous, diffuse headache. Since several months, she noted double vision during episodes of tiredness. She also realised a depressive mood.

**Figure 1:**
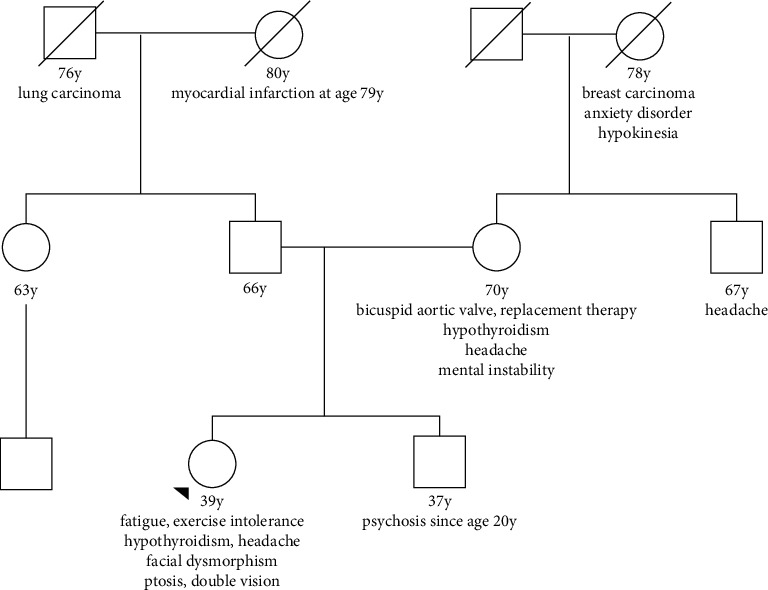
Pedigree of the index patient's family revealing that a maternal trait of inheritance is quite likely despite absence of the culprit variant in the mother.

Her family history was positive for hypothyroidism, aortic valve replacement therapy at age 52 y because of a bicuspid aortic valve, headache, hypothyroidism, and mental instability in her mother, psychosis, and exercise intolerance in her brother, breast cancer, anxiety disorder, and fatigability in her grandmother from the mother's side (Figure 1).

Clinical neurologic exam at age 28 y revealed short stature, bilateral ptosis, hypertelorism, reduced tendon reflexes on the upper limbs, reduced patella tendon reflexes on the lower limbs, and gnome calves.

Workup for a suspected MID revealed lactic acidosis, ragged-red fibers on muscle biopsy, mild complex-I deficiency (complex-1 activity: 27 mU/mU CS (*n*, 28–76 mU/mU CS), and the variant m.3243A>G with a heteroplasmy rate of 70% in muscle. The mutation was not detected in blood or urinary epithelial cells. The mutation could not be detected in blood or urine of her mother either. Cerebral magnetic resonance imaging (MRI), electrocardiography (ECG), and echocardiography were noninformative. She was recommended to regularly take riboflavin (300 mg/d) and coenzyme-Q (300 mg/d) in addition to L-thyroxin with limited benefit.

## 3. Discussion

The case shows that fatigue and exercise intolerance can be the initial phenotypic manifestation of a MID besides short stature. The case also demonstrates that the disease course of a MID can be slowly progressive over years with only delayed affection of organs other than the muscle. Extramuscular manifestations in the index patient were headache, hypothyroidism, and short stature. Lactic acidosis most likely derived from affection of the skeletal muscles.

Fatigue is a nonspecific term with many different meanings. Fatigue may have an organic or a psychological cause. Among the organic causes, fatigue may derive from a genetic or an acquired defect. Fatigue may be present already without any physical activity or only in association with physical exercise. The latter is also known as exercise intolerance or exhaustibility. Acquired causes of fatigue include disorders of the central nervous system, the peripheral nervous system, the muscles, endocrinologic disorders, cardiac disease, pulmonary dysfunction, renal insufficiency, infectious diseases, immunological diseases, malignancy, malnutrition, or drugs. Among the psychological causes, fatigue may derive from depression, chronic pain, chronic overstimulation, or chronic overload (e.g., seafarer fatigue).

Since the brain, heart, and kidneys were not affected in the index patient, since substitution of hypothyroidism did not resolve fatigue, and since there was no systemic disease, fatigue and exercise intolerance were most likely due to affection of the skeletal muscles. Whether lactic acidosis contributed to fatigue or was the sole cause of fatigue remains speculative. Since lactic acidosis was only mild, it was most likely not responsible for fatigue and exercise intolerance. More likely, the initial manifestations were caused by the mismatch between energy requirements and energy production. A contributing factor to fatigue in the index patient could be the depression. Nocturnal hypoventilation was excluded as a cause of fatigue as polysomnography including oximetry did not reveal desaturations overnight.

Fatigue is a common phenotypic feature of MIDs and has been reported in various types of syndromic and nonsyndromic MIDs, irrespective of an mtDNA or nDNA mutation as the underlying cause, and irrespective if the respiratory chain or other metabolic pathways (e.g. urea cycle, ketone body metabolism, and heme synthesis), receptors, transport proteins, pore proteins, or signalling mitochondrial proteins were involved [2]. In a study of 48 adults with a MID, 71–100% reported fatigue depending on the measure used, and the correlation between severity of fatigue and severity of disease [2]. In a study of 72 MID patients carrying the m.3243A>G variant, 60% showed a high degree of fatigue as assessed by the RAND-SF36 score [3]. In a case series of four patients carrying the m.3243A>G variant, one patient manifested exclusively with exercise intolerance, muscle aching since childhood, and mental and physical fatigue [4]. Unfortunately, heteroplasmy rates were not provided and it was not mentioned which of the manifestations occurred first [4]. Usually, fatigue is one among other features of the m.3243A>G phenotype at onset or develops later in the course [5].

Exercise intolerance is also a frequent manifestation of MIDs or m.3243A>G carriers but usually occurs in association with other phenotypic features [6]. In a study of 47 Chinese families carrying the m.3243A>G variant, the most frequent manifestations in the index patients' mothers were lactic acidosis, exercise intolerance, short stature, and weight loss [7]. In a study of 82 Dutch m.3243A>G carriers, exercise intolerance was reported in 54% of them, being thus the most frequent manifestation after hearing loss and gastrointestinal compromise [8]. In a study of 11 MID patients of whom 5 carried the m.3243A>G variant, a single patient manifested exclusively with exercise intolerance [9]. In a patient with only mild exercise intolerance, normal muscle histochemistry, and normal respiratory chain activity in vitro, the heteroplasmy rate of the variant m.3243A>G was 6% [10]. Despite this low heteroplasmy rate, the maximum rate of ATP production was reduced to 35% in his calf muscles [10].

Despite the absence of the causative mtDNA variant in the index patient's mother in the blood and urine, the variant was regarded as inherited given the clinical presentation of her mother and grandmother from the mother's side and the putative maternal trait of inheritance.

## 4. Conclusions

This case shows that the initial manifestation of a MID can be fatigue and exercise intolerance, that MIDs due to the m.3243A>G variant may have a slowly progressive course and only delayed multisystem involvement, that the variant m.3243A>G may not only manifest as syndromic MID, particularly MELAS, but also as nonsyndromic phenotype, and that MIDs should be considered as differentials of chronic fatigue even if no other phenotypic manifestation of the MID is present.

## Data Availability

Clinical data were used to support this study.

## References

[1] Kanungo S., Morton J., Neelakantan M., Ching K., Saeedian J., Goldstein A. (2018). Mitochondrial disorders. *Annals of Translational Medicine*.

[2] Parikh S., Galioto R., Lapin B. (2019). Fatigue in primary genetic mitochondrial disease: no rest for the weary. *Neuromuscular Disorders*.

[3] Verhaak C., de Laat P., Koene S. (2016). Quality of life, fatigue and mental health in patients with the m.3243A>G mutation and its correlates with genetic characteristics and disease manifestation. *Orphanet Journal of Rare Diseases*.

[4] Blum S., Robertson T., Klingberg S., Henderson R. D., McCombe P. (2011). Atypical clinical presentations of the A3243G mutation, usually associated with MELAS. *Internal Medicine Journal*.

[5] Komlósi K., Kellermayer R., Maász A. (2005). Maternally inherited deafness and unusual phenotypic manifestations associated with A3243G mitochondrial DNA mutation. *Pathology and Oncology Research*.

[6] Gál A., Szabó A., Pentelényi K., Pál Z. (2008). Maternally inherited diabetes mellitus, deafness, chronic progressive external ophthalmoplegia and myopathy as the result of A3243G mutation of mtDNA. *Orvosi Hetilap*.

[7] Ma Y., Fang F., Cao Y. (2010). Clinical features of mitochondrial DNA m.3243A>G mutation in 47 Chinese families. *Journal of the Neurological Sciences*.

[8] de Laat P., Koene S., van den Heuvel L. P. W. J., Rodenburg R. J. T., Janssen M. C. H., Smeitink J. A. M. (2012). Clinical features and heteroplasmy in blood, urine and saliva in 34 dutch families carrying the m.3243A>G mutation. *Journal of Inherited Metabolic Disease*.

[9] Lu Y., Zhao D., Yao S. (2017). Mitochondrial tRNA genes are hotspots for mutations in a cohort of patients with exercise intolerance and mitochondrial myopathy. *Journal of the Neurological Sciences*.

[10] Chinnery P. F., Taylor D. J., Brown D. T., Manners D., Styles P., Lodi R. (2000). Very low levels of the mtDNA A3243G mutation associated with mitochondrial dysfunction in vivo. *Annals of Neurology*.

